# Massive subcutaneous emphysema after traumatic pneumothorax

**DOI:** 10.1002/ccr3.2311

**Published:** 2019-07-15

**Authors:** Nikolaos Machairas, Anna Paspala, Athanasios Syllaios, Dimitrios Schizas

**Affiliations:** ^1^ Third Department of Surgery, Attikon University Hospital National and Kapodistrian University of Athens Athens Greece; ^2^ First Department of Surgery, Laikon Hospital National and Kapodistrian University of Athens Athens Greece

**Keywords:** chest tube, massive, pneumothorax, subcutaneous emphysema, traumatic

## Abstract

A simple case/asymptomatic pneumothorax not deemed to necessitate drainage can quickly change, and patient safety can be compromised. Chest tube insertion with increased suction is considered a safe and efficient strategy in patients with extensive subcutaneous emphysema following traumatic pneumothorax.

## 1 INTRODUCTION

**Figure 1:**
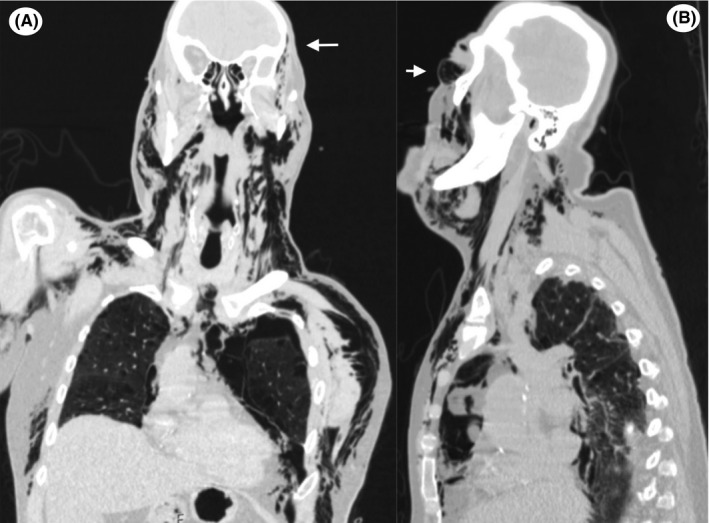
A, Computed tomography scan demonstrating the extension of the emphysema up to the temporal subcutaneous tissue. B, Computed tomography (orbital view)

An 81‐year‐old man presented to our emergency department after falling from an escalator 2 hours earlier. He complained of mild dyspnea and left‐sided chest pain. On physical examination, he was hemodynamically stable with blood pressure of 105/65 mm Hg and a heart rate of 95 beats per minute. Chest examination revealed left chest hypoventilation with associated hypoxemia (Sp02 = 92%). Full body scan showed a linear left pneumothorax (<10% volume) and two isolated rib fractures. No surgical intervention was deemed essential. Two hours following admission, he presented increasing dyspnea and a swelling that had rapidly progressed from the left chest up to the cervicofacial area. Head and chest computed tomography revealed an increased left pneumothorax, pneumomediastinum, and massive subcutaneous emphysema which extended up to the temporal (Figure 1A) and orbital (Figure 1B) subcutaneous tissue. He was immediately treated with chest drainage. Following the insertion of the thoracic tube, the patient was thoroughly evaluated for rupture of the trachea or esophagus with bronchoscopy and esophagoscopy that did not reveal any obvious perforation. Two days postadmission, swelling, dyspnea, and subcutaneous emphysema progressively improved. The patient was discharged after 6 days in a good condition. Ten days after discharge, both the emphysema and pneumothorax had completely resolved. One year after the incident, there is a complete resolution of the patient's symptoms as well as complete clinical and radiological resolution of the pneumothorax and emphysema.

This report illustrates wonderfully how a simple case/asymptomatic pneumothorax not deemed to necessitate drainage can quickly change and patient safety can be compromised. Chest tube insertion with increased suction is considered a safe and efficient strategy in such clinical situations. A wait‐and‐see strategy is advised in patients with limited subcutaneous emphysema, whereas additional drainage may contribute to faster patient relief. All in all, such interventions must be tailored on an individual basis.^1^, ^2^

## CONFLICT OF INTEREST

The authors declare no conflict of interest.

## AUTHORS' CONTRIBUTIONS

NM and AP collected the data, wrote the manuscript, and revised the manuscript. AS wrote the manuscript and revised the manuscript. DS wrote the manuscript, revised the manuscript, provided intellectual input, and supervised the manuscript.
